# Morphometry of Conus Medullaris in the Saudi Population and Its Clinical Importance

**DOI:** 10.7759/cureus.32279

**Published:** 2022-12-07

**Authors:** Sanket D Hiware, Christopher A Vallaba Doss, Hossain A Alola, Maha M Alshahrani, Abdulhadi A Alamri, Mohammed H Aljohani, Yasir D Aldulaijan, Ali Z Alawami, Abdelmohsen W Nasser, Abdullah S Alhashim, Kamaluddin H Motawei

**Affiliations:** 1 Anatomy, Imam Abdulrahman Bin Faisal University, Dammam, SAU; 2 Statistics/Biostatistics, Imam Abdulrahman Bin Faisal University, Khobar, SAU; 3 Radiology, Imam Abdulrahman Bin Faisal University, Dammam, SAU; 4 Radiology, King Fahd University Hospital, Khobar, SAU

**Keywords:** morphometry, magnetic resonance imaging, spinal cord, intervertebral disc space, conus medullaris

## Abstract

Objective

The objective of this article is to evaluate the morphometry and termination of conus medullaris (CM) in the eastern province of Saudi Arabia.

Methodology

The lumbar spine magnetic resonance imaging (MRI) scans of 179 citizens of Saudi Arabia were selected and divided into males and females. Parameters such as the level of termination of CM, the length/width of CM, and its termination were reported.

Results

The maximum number of males and females were observed in the less than 20 years age group as 23 (25.5%) and 22 (24.7%), respectively. The termination level of CM was at the (first lumbar vertebra) L1 vertebra in 51.4% of the Saudi population. The level of termination of CM is below the (second lumbar vertebra) L2 level in one patient at L3, L3-L4, L5, and L5-S1 levels. On comparing the length of CM, no statistically significant difference was observed between the different levels of the vertebra. However, there was a statistically significant difference between the width and the CM termination (p=0.02). The various age groups and CM termination had no statistically significant correlation (p=0.47).

Conclusion

The most common level of termination of conus medullaris was at the L1 vertebra. The level of CM termination is strongly associated with the width of CM.

## Introduction

The upper two-thirds of the vertebral canal's length is occupied by the spinal cord. At the level of the foramen magnum boundary, it continues cranially as the medulla oblongata. Caudally, the spinal cord terminates as an expanded structure called conus medullaris (CM) [1]. There is variation in the termination of CM in different races [1,2]. The level of CM termination varies from the twelfth thoracic level (T12) to the third lumbar (L3). CM has an oval shape and a ventral groove at the (eleventh thoracic level) T11 level [2].

During fetal development, the total length of the vertebral canal is occupied by the spinal cord. The spinal nerves leave the vertebral canal perpendicular to the spinal cord through the intervertebral foramina. The degree of termination of the CM changes as a result of the vertebral column's growth, exceeding the spinal cord. Due to the increasing distance between the lumbosacral cord segments and their equivalent vertebrae, spinal nerves leaving the vertebral canal from these regions become gradually longer and vertical. The spinal nerve emerging from the lumbosacral enlargement and CM is collectively called the cauda equine (CE) [3]. The CM and CE together give rise to spinal nerves that provide motor and sensory supply to the lower extremity, pelvis, and perineum. It also provides parasympathetic innervation to the pelvic viscera [3,4].

Conus medullaris syndrome refers to a group of symptoms brought by damage to the CM [4]. Radicular discomfort, bowel/bladder malfunction, patchy sensory loss (saddle anesthesia), and muscle weakness (lower extremity) at the level of the lumbar and sacral roots are all signs of cauda equina syndrome [5]. This syndrome may be linked to a Chiari malformation in pediatric patients. In such patients, the conus medullaris ends at or below the L2-L3 lumbar vertebrae disc space [5,6]. This study evaluated the morphometry and termination of the conus medullaris in the eastern province of Saudi Arabia.

## Materials and methods

The present study was retrospective and performed in the Department of Anatomy and Department of Radiology of Imam Abdulrahman Bin Faisal University in Saudi Arabia. In this study, 179 lumbar spine magnetic resonance imaging (MRI) scans of patients were studied. The lumbar spine MRIs were done using a 1.5 Tesla MRI machine (Siemens, Munich, Germany), with a thickness of every slice was 3 millimeters (mm). After placing the patients in the supine position, mid-sagittal T1 and T2 weighted images were acquired. Data from Digital Imaging and Communications in Medicine (DICOM, developed by the American College of Radiology) was used to transfer all pictures to the computer. The conus medullaris tip can be observed on midline sagittal T1 and T2-weighted MRI.

The study was done using a convenient sampling technique. The study includes 179 lumbar spine MRIs, whereas the exclusion criteria included were comorbid neurological disease, including neuropathy or cerebral infarction, a known history of brain/spinal surgery, intermittent claudication, symptoms of sensory or motor diseases like gait disturbances, clumsiness, numbness, or motor weakness, spinal infection, past history of any fracture in a vertebra, rheumatoid arthritis, severe low back pain, congenital anomalies or degenerative diseases of the vertebral body, autoimmune diseases, malignancies or previous operative surgery at the lumbosacral area, or chronic renal failure [6]. Furthermore, full-length, free-standing spinal radiographs with fists on the clavicles were obtained from all the participants. All the images were transferred to a computer as DICOM data. 

Parameters such as the level of termination of CM, the length/width of CM, and its termination were assessed. The data were entered in a Microsoft Excel sheet (Microsoft, Redmond, Washington) and analyzed using SPSS software version 21 (IBM Inc. Armonk, New York). Statistical analysis was performed using the Chi-square test, and a p-value of <0.05 was considered statistically significant.

## Results

A total of 179 lumbar spine MRI scans were studied. Table 1 shows that there were 90 males and 89 females in the study. The maximum number of males and females were observed in the less than 20 years age group as 23 (25.5%) and 22 (24.7%), respectively.

**Table 1:** Distribution of patients

Age groups	Male (%)	Female (%)	Total (%)
<20 years	23 (25.5)	22 (24.7)	45 (25.1)
21-40 years	20 (22.2)	20 (22.5)	40 (20.7)
41-60 years	20 (22.2)	20 (22.5)	40 (22.3)
61-80 years	21 (22.4)	21 (23.6)	42 (23.5)
>80 years	6 (6.7)	6 (3.9)	12 (6.7)
Total	90	89	179

Table 2 depicts that the most common level of termination of CM is at the L1 vertebra, which was observed in 92 (51.4 %) subjects. The level of termination of CM at T12-L1, L1-L2, and L2 levels was observed in 25 (14%), 24 (13.4%), and 27 (15.1%) of the cases. Only one patient in each group was observed to have level of termination of CM at L3, L3-L4, L5, and L5-S1 levels.

**Table 2:** Level of termination of CM CM - conus medullaris

Level	Number	Percentage
T12	7	3.9
T12-L1	25	14.0
L1	92	51.4
L1-L2	24	13.4
L2	27	15.1
L3	1	0.6
L3-L4	1	0.6
L5	1	0.6
L5-S1	1	0.6

Table 3 shows that there was no statistically significant difference between the length of CM at different levels (p=0.13). On comparing the width of conus medullaris from T12 to L5-S1 level, a significant difference was observed among them (p=0.02).

**Table 3:** Comparison between the length and width of conus medullaris at different levels *p<0.05; Data presented as mean ± standard deviation

Variables	Conus medullaris	Number	Mean	p-value
Length (in millimeters)	T12	7	16.21 ± 3.67	0.13
	T12-L1	25	20.09 ± 6.01	
	L1	92	19.46 ± 5.62	
	L1-L2	24	20.18 ± 5.48	
	L2	27	19.91 ± 5.40	
	L3	1	9.50 ± 0.0	
	L3-L4	1	12.10 ± 0.0	
	L5	1	9.90 ± 0.0	
	L5-S1	1	12.40 ± 0	
Width (in millimeters)	T12	7	5.79 ± 0.93	0.02*
	T12-L1	25	5.37 ± 0.98	
	L1	92	5.05 ± 0.69	
	L1-L2	23	5.17 ± 0.86	
	L2	27	4.81 ± 0.87	
	L3	1	4.20 ± 0	
	L3-L4	1	4.00 ± 0	
	L5	1	3.60 ± 0	
	L5-S1	1	5.00 ± 0	

Table 4 compares the association between different age groups and the termination of conus medullaris. It was observed that no statistical significance was observed in correlating different age groups with CM termination (p=0.47).

**Table 4:** Comparison between the different age groups with CM termination CM - conus medullaris

Level	Age groups (years)						Chi-squared test	p-value
	<20, N (%)	21- 40, N (%)	41-60, N (%)	61-80, N (%)	>80, N (%)	Total, N (%)		
T12	1 (0.6)	2 (1.1)	2 (1.1)	2 (1.1)	0 (0.0)	7 (3.9)	31.83	0.47
T12-L1	5 (2.8)	7 (3.9)	8 (4.5)	4 (2.2)	1 (0.6)	25 (14.0)		
L1	19 (10.6)	16 (8.9)	21 (11.7)	26 (14.5)	10 (5.6)	92 (51.4)		
L1-L2	12 (6.7)	3 (1.7)	5 (2.8)	3 (1.7)	1 (0.6)	24 (13.4)		
L2	4 (2.2)	9 (5.0)	4 (2.2)	7 (3.9)	3 (1.7)	27 (15.1)		
L3	1 (0.6)	0 (0.0)	0 (0.0)	0 (0.0)	0 (0.0)	1 (0.6)		
L3-L4	1 (0.6)	0 (0.0)	0 (0.0)	0 (0.0)	0 (0.0)	1 (0.6)		
L5	1 (0.6)	0 (0.0)	0 (0.0)	0 (0.0)	0 (0.0)	1 (0.6)		
L5-S1	1 (0.6)	0 (0.0)	0 (0.0)	0 (0.0)	0 (0.0)	1 (0.6)		

## Discussion

The conus is observed at the level of L1-L2 intervertebral space once a child is two years of age [7]. The apex of the conus is regarded as the landmark for termination within the spinal canal, and the CM tip is easily identifiable on midline sagittal T1-weighted magnetic resonance imaging [8]. According to numerous earlier research using both cadavers and live people, the level of CM in the general population of children and adults ranges between T12 to L3 [5,8,9].

In this study, 90 males and 89 females were studied. The maximum number of males and females were observed in the less than 20 years age group as 23 (25.5%) and 22 (24.7%), respectively. However, in our study, 179 lumbar spine MRIs were evaluated. Demiryurek et al. evaluated the level of termination of conus medullaris according to age and sex [10]. This retrospective study used a 0.5 Tesla magnetic resonance imaging machine and a total of 600 participants were chosen who had no spinal canal disease on the lumbar MR imaging test. In the study group, the level of conus medullaris was primarily seen at the T12-L1 intervertebral disc level. With increasing age, no noticeable difference in the conus level was observed. The findings imply that there was no difference in the level of conus with increasing age in the adult population. 

The L1 vertebra (51.4%) is the most frequent level of termination of CM, and this finding was statistically significant. The level of termination of CM below the L2 level was observed in only one patient in each group. Moon et al. used the MRI method on 189 Korean patients aged between two to 94 years, including 93 men and 94 women, to study the level of termination of CM [11]. There were no subjects from other racial or ethnic groups. The L1 bodies made up the majority of the conus medullaris' tip from the upper T12 body to the L2-L3 disc, followed by the L2 bodies (23.4%), the L1-L2 disc, and the L2-L3 disc (2.1%). Conus shapes were categorized as type A in 76 patients (39.6%), type B in 59 patients (31%), and type C in 54 patients (29.4%). Theoretically, it is speculated that type C conus experiences the least bruises by the accidentally-retropulsed bone fragments in burst fractures, whereas type A conus is assumed to be most easily bruised, with type B being affected to an intermediate level. In none of the patients did the CM terminate at the L3 body level.

The study shows that a statistically significant difference between the length of CM at different levels was not observed. However, on comparing the width of conus medullaris from T12 to L5-S1 level, a statistically significant difference was observed among them.

In the study, the average length of CM at L1, L2, T12-L1, and L1-L2 was 19.46 ± 5.62, 19.91 ± 5.40, 20.09 ± 6.01, and 20.18 ± 5.48 millimeters, respectively. However, statistical difference was observed on comparing the average breadth of CM at L1, L2, T12-L1, and L1-L2 were 5.05 ± 0.69, 4.81 ± 0.87, 5.37 ± 0.98, and 5.37 ± 0.98 millimeters, respectively. Malas et al. examined the variations between the termination level of the conus and the termination level of the largest portion of the transverse diameter of the lumbosacral enlargement during the period of fetal development as well as adulthood in 80 cases, including 20 fetuses, 30 neonates, and 30 adults [12]. Our findings are coherent with the study conducted by Malas et al. with respect to the breadth of CM and its level of termination [12]. For fetuses, they employed dissection techniques; for premature infants, they used ultrasonography; and for adults, MR imaging was utilized. For fetuses, the conus level was between L3 and L1; for premature babies, it was between L1 and L3; and for adults, it was between T12 and L2.

In the study, the level of CM termination was compared with the different age groups. Most of the patients had a termination at the L1 level, with 26 (14.5%) of patients between 61-80 years of age. In one patient in the younger age group (less than 20 years), the level of termination of CM was observed at L3, L3-L4, L5, and L5-S1 levels. However, a literature search revealed that no such comparison was made with the level of termination of CM in different age groups.

One of the limitations of the study is the small sample size. The data could be better speculated in case if the study was done on more lumbar MRI scans. Another limitation of the study was that we didn't study the influence of age and sex on the position of the conus medullaris.

## Conclusions

The study didn't find any association between the age group with CM termination. The most common level of termination of conus medullaris was at the L1 vertebra. The level of conus medullaris termination is strongly associated with the width of CM. However, the study depicted that the length of conus medullaris has no association with its level of termination.
